# FDG-PET/MRI for Nonoperative Management of Rectal Cancer: A Prospective Pilot Study

**DOI:** 10.3390/tomography8060227

**Published:** 2022-11-09

**Authors:** Semra Ince, Malak Itani, Lauren E. Henke, Radhika K. Smith, Paul E. Wise, Matthew G. Mutch, Sean C. Glasgow, Matthew L. Silviera, Katrina S. Pedersen, Steven R. Hunt, Hyun Kim, Tyler J. Fraum

**Affiliations:** 1 Department of Radiology, Washington University School of Medicine, Saint Louis, MO 63110, USA; 2 Department of Radiation Oncology, Washington University School of Medicine, Saint Louis, MO 63110, USA; 3 Section of Colon and Rectal Surgery, Department of Surgery, Washington University School of Medicine, Saint Louis, MO 63110, USA; 4 Division of Oncology, Department of Medicine, Washington University School of Medicine, Saint Louis, MO 63110, USA

**Keywords:** rectal cancer, nonoperative management, FDG-PET/MRI

## Abstract

Nonoperative management (NOM) is increasingly utilized for rectal cancer patients with a clinical complete response (cCR) following total neoadjuvant therapy (TNT). The objective of this pilot study was to determine whether FDG-PET/MRI alters clinical response assessments among stage I-III rectal cancer patients undergoing TNT followed by NOM, relative to MRI alone. This prospective study included 14 subjects with new rectal cancer diagnoses. Imaging consisted of FDG-PET/MRI for initial staging, post-TNT restaging, and surveillance during NOM. Two independent readers assessed treatment response on MRI followed by FDG-PET/MRI. Inter-reader differences were resolved by consensus review. The reference standard for post-TNT restaging consisted of surgical pathology or clinical follow-up. 7/14 subjects completed post-TNT restaging FDG-PET/MRIs. 5/7 subjects had evidence of residual disease and underwent total mesorectal excision; 2/7 subjects had initial cCR with no evidence of disease after 12 months of NOM. FDG-PET/MRI assessments of cCR status at post-TNT restaging had an accuracy of 100%, compared with 71% for MRI alone, as FDG-PET detected residual tumor in 2 more subjects. Inter-reader agreement for cCR status on FDG-PET/MRI was moderate (kappa, 0.56). FDG-PET provided added value in 82% (9/11) of restaging/surveillance scans. Our preliminary data indicate that FDG-PET/MRI can detect more residual disease after TNT than MRI alone, with the FDG-PET component providing added value in most restaging/surveillance scans.

## 1. Introduction

Colorectal cancer is the third most frequent cancer globally, with rectal cancer accounting for one in three cases [1]. Except for a small group of favorable-risk stage I tumors, the standard therapy of stage I-III rectal cancer traditionally includes total mesorectal excision (TME), with or without neoadjuvant chemoradiation and adjuvant chemotherapy. TME in the form of an abdominoperineal resection (APR) results in a permanent stoma, whereas TME in the form of a low anterior resection (LAR) obviates the need for a permanent ostomy but often adversely affects bowel function. Both procedures can be associated with considerable perioperative morbidity, which often limits the number of patients who successfully receive all intended adjuvant chemotherapy to reduce risk of distant metastatic relapse. Recently, the application of both radiation and full systemic chemotherapy prior to surgery (total neoadjuvant therapy, TNT) has enhanced clinical and pathologic complete response rates and produced durable long-term disease-free survival in selected patients with rectal adenocarcinoma, even without TME [2].

Consequently, nonoperative management (NOM) is an increasingly utilized treatment strategy for selected patients who achieve a clinical complete response (cCR) after TNT, with low rates of local recurrence at 12 months post-treatment [3,4,5,6]. Maximizing imaging sensitivity for residual or recurrent disease is critical to ensuring that cCR is accurately identified and that NOM failures are detected early, when surgery is still feasible and potentially curative. Despite the high accuracy of pelvic MRI for initial staging, its ability to predict the presence and extent of tumor following neoadjuvant chemoradiation is limited, even when dedicated MRI-based tumor regression scoring systems are used [7]. Positron emission tomography (PET) utilizing the glucose analogue 2-deoxy-2-[^18^F] fluoro-D-glucose (FDG), which has been shown to predict disease-free survival in patients with locally advanced rectal cancer treated with neoadjuvant chemoradiation, might be complementary to MRI for treatment response assessment [8].

To this point, a recent study suggested that FDG-PET/MRI may be more accurate than MRI alone for predicting residual viable tumor among rectal cancer patients undergoing TME following neoadjuvant therapy [9]. However, the utility of FDG-PET/MRI for response assessment in the NOM setting has not been established. Thus, the objective of this pilot study was to determine whether FDG-PET/MRI has the potential to alter clinical response assessments among rectal cancer patients undergoing TNT followed by NOM, relative to MRI alone.

## 2. Materials and Methods

### 2.1. Subjects

This IRB-approved, HIPAA-compliant prospective study enrolled patients with newly diagnosed rectal cancer referred to the colorectal surgery clinic at our tertiary care center between June 2019 and September 2021. Inclusion criteria were as follows: (a) at least 18 years of age; (b) biopsy-proven rectal adenocarcinoma with clinically suspected or established stage I-III (cT1-4, N0-2, M0) disease; (c) anticipated TNT as part of a NOM treatment strategy; and (d) ability to provide written informed consent. Exclusion criteria were as follows: (a) prior surgical resection of rectal cancer (endoscopic or TME), (b) contraindication to MRI or gadolinium-based contrast; (c) comorbidities limiting ability to cooperate with a PET/MRI examination; and (d) end-stage renal disease.

### 2.2. Imaging and Clinical Management Algorithm

All enrolled subjects underwent FDG-PET/MRI of the pelvis and contrast-enhanced CT of the chest and abdomen for initial staging. Subjects found to have metastatic disease (i.e., stage IV) were not eligible to continue on the study. If deemed to be immediate surgical candidates (based on the initial clinical staging, as well as other patient-specific factors), subjects underwent TME, at which point all study-related imaging ceased. Otherwise, subjects received TNT consisting of short-course radiation therapy (i.e., 25 Gy in 5 fractions) followed by consolidation chemotherapy (mFOLFOX6 or CAPOX). Chemotherapy was initiated 2–4 weeks after completion of radiation therapy and continued for 15–16 weeks, depending on the regimen selected. Assessment of treatment response occurred approximately 1 month after the completion of chemotherapy and consisted of restaging imaging (i.e., FDG-PET/MRI [F/U #1], contrast-enhanced CT of the chest and abdomen), lower endoscopy (with or without biopsy, depending on findings), and digital rectal exam (DRE). Note that any residual mucosal ulceration, nodularity, or irregularity on lower endoscopy at F/U #1 was considered a possible indication of residual disease and prompted biopsy. Subjects with clinically suggested residual disease (based on multidisciplinary discussion) or biopsy-proven residual disease underwent TME, at which point all study-related imaging ceased. Subjects deemed to have a cCR were placed on a NOM surveillance regimen performed every 3 months, consisting of serum carcinoembryonic antigen (CEA) levels, lower endoscopy (with or without biopsy), DRE, and follow-up imaging. Follow-up imaging consisted of contrast-enhanced CT of the chest and abdomen paired with either pelvic MRI alone (3 mos, 9 mos) or FDG-PET/MRI (6 mos [F/U #2], 12 mos [F/U #3]).

### 2.3. FDG-PET/MRI Imaging Protocol

Subjects fasted for at least 4 h prior to their imaging appointments. After confirmation of blood glucose levels below 200 mg/dL, FDG was administered intravenously according to our institution’s standard weight-based scale. At 55–65 min post-injection, patients entered the imaging suite and received 1 mg of glucagon intravenously, immediately followed by the initiation of PET/MRI. The PET component consisted of a 30 min list mode acquisition at a single pelvic station, centered craniocaudally on the rectal tumor. The 30 min of PET data was reconstructed as a single static image and as a dynamic series with ten 3 min frames. The reconstruction parameters were as follows: 3D, OP-OSEM, 3 iterations, 21 subsets, 172 × 172 matrix, zoom of 1, 4 mm Gaussian filter.

The MRI component, which was also limited to the pelvis, consisted of the following sequences, which adhered to the standard technical recommendations espoused by the Society of Abdominal Radiology disease-focused panel (SAR DFP) on rectal and anal cancer [10]: (a) two-point Dixon (attenuation correction) in the axial plane; (b) high spatial resolution small field-of-view (FOV) T2-weighted turbo spin echo in the sagittal, oblique axial, and oblique coronal planes; (c) large FOV T2-weighted single-shot fast spin echo in the axial and coronal planes; (d) diffusion-weighted imaging (*b* values: 50, 500, 1000 s/mm^2^); (e) pre-contrast 3D T1-weighted gradient echo in the axial plane; and post-contrast 3D T1-weighted gradient echo in the axial, coronal and sagittal planes. For post-contrast imaging, gadoterate meglumine (Dotarem; Guerbet LLC, Princeton, NJ, USA) was administered intravenously according to our institution’s standard weight-based protocol.

### 2.4. Image Review and Analysis

Two readers (Reader 1 and Reader 2) with knowledge of the subject’s clinical histories but not pathology results or clinical follow-up independently interpreted the MRI component, followed by the PET component (including fused images as deemed appropriate). Both readers were dual-trained in diagnostic radiology and nuclear medicine, with subspecialty expertise in abdominopelvic MRI and PET. Interpretive differences were resolved by consensus review. At the restaging and surveillance time points, readers did not have access to results of the DRE or lower endoscopy (with or without biopsy), as these assessments were performed after the imaging. However, the readers did have access to prior PET/MRI and MRI examinations for assessment of changes over time. MRI and PET interpretations were separately documented in the subject’s medical record and available to the multidisciplinary team managing the subject’s oncologic care. Reader 1 extracted maximum SUVs from the PET images.

At the first restaging and all subsequent follow-up time points, both readers utilized an established MRI-based tumor regression grade (mrTRG) system for assessment of treatment response on the MRI component [11]:

- mrTRG 1: no/minimal fibrosis visible (tiny linear scar) and no tumor signal
- mrTRG 2: dense fibrotic scar but no macroscopic tumor signal
- mrTRG 3: fibrosis predominates but obvious measurable areas of tumor signal
- mrTRG 4: tumor signal predominates with little/minimal fibrosis
- mrTRG 5: tumor signal only (no fibrosis) or tumor progression

The mrTRG system is based on interpretation of T2-weighted images only, but readers were allowed to utilize other MRI sequences (e.g., diffusion-weighted imaging) to assist in interpretation of the T2-weighted images.

Following mrTRG assignment, readers reviewed the PET images, including fused series as needed. Both readers then assigned a novel PET/MRI-based tumor regression grade (pmrTRG) developed at our institution for assessment of treatment response on the MRI and PET components:

- pmrTRG 1: mrTRG 1–2 and no appreciable tumor signal on PET (FDG uptake in rectal wall similar to or less than background rectum)
- pmrTRG 2: mrTRG 1–2 and possible tumor on PET (focal FDG uptake in wall of rectum equivocally above background rectal wall)
- pmrTRG 3: mrTRG 3–5 or definite tumor on PET (focal FDG uptake in wall of rectum clearly above background rectal wall)

Readers also documented whether the addition of the PET component provided any added value relative the MRI component alone and, if so, how the PET component was helpful or changed their overall assessments of disease status.

### 2.5. Statistical Analysis

All statistical analyses were performed in Excel 2016 (Microsoft; Redmond, WA, USA). Subject ages, demographics, and tumor/clinical attributes were summarized descriptively. For the inter-reader agreement analysis, Cohen’s kappa was calculated for mrTRG and pmrTRG per standard ordinal categories, as well as in a binary fashion as follows: mrTRG 1–2 (no evidence of residual tumor) vs. 3–5 (evidence of residual tumor). A pmrTRG of 2–3 was considered positive for residual disease. Agreement was assessed according to the following scale: 0.01–0.20 = slight; 0.21–0.40 = fair; 0.41–0.60 = moderate; 0.61–0.80 = substantial; 0.81–1.00 = almost perfect. Diagnostic accuracy was calculated according to the following equation: (TP+TN)/(TP+TN+FP+FN), where TP = true positive, TN = true negative, FP = false positive, and FN = false negative.

## 3. Results

**Figure 1:**
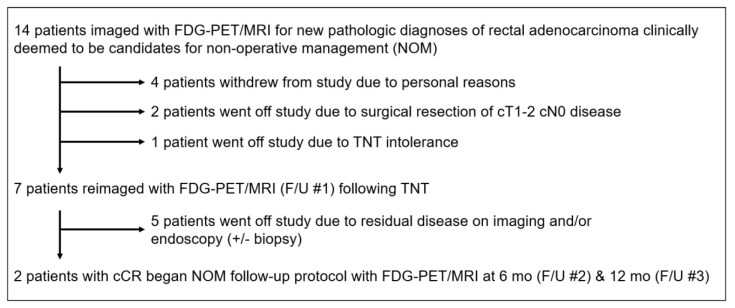
Enrollment schema. Abbreviations: TNT = total neoadjuvant therapy.

This study enrolled 14 subjects (7 male, 7 female) with a median age of 51.6 years (range, 38.4–83.3 years). Tumor characteristics from initial staging FDG-PET/MRI of the pelvis are shown in Table 1. Among these 14 subjects, 7 were reimaged with FDG-PET/MRI after TNT (Figure 1). Of these 7 patients, 2 (Subject #1, Subject #6) were deemed to have a cCR by study criteria and began a NOM follow-up protocol. For these 2 patients, clinical follow-up (CEA levels, imaging, endoscopy +/− biopsy) for 12 months after the first restaging FDG-PET/MRI (F/U #1) was the reference standard. The remaining 5 patients had evidence of residual disease on the restaging FDG-PET/MRI [F/U #1] and underwent TME, so surgical pathology was the reference standard for these subjects.

Initial staging information and post-TNT assessments of disease status (including mrTRG, pmrTRG, primary tumor status, and nodal status) are shown in Table 2 for both readers and for the two-reader consensus. According to consensus reads, 3 of 7 subjects had evidence of residual disease on MRI alone (i.e., mrTRG 3–5), whereas 5 of 7 subjects had evidence of residual disease on FDG-PET/MRI (i.e., pmrTRG 2–3). For all subjects with suspected residual disease on MRI and/or FDG-PET, the suspicious finding was at the primary tumor site rather than in the regional lymph nodes. Only 3 of 7 subjects had evidence of residual disease on endoscopy (with or without biopsy) at the initial restaging (F/U #1) time point. For the mrTRG consensus read, there were 2 FNs, 2 TNs, and 3 TPs, for an overall accuracy of 71%. For the pmrTRG consensus read, there were 2 TNs and 5 TPs, for an overall accuracy of 100%. For endoscopy +/− biopsy, there were 2 FNs, 2 TNs, and 3 TPs, for an overall accuracy of 71%.

**Figure 2:**
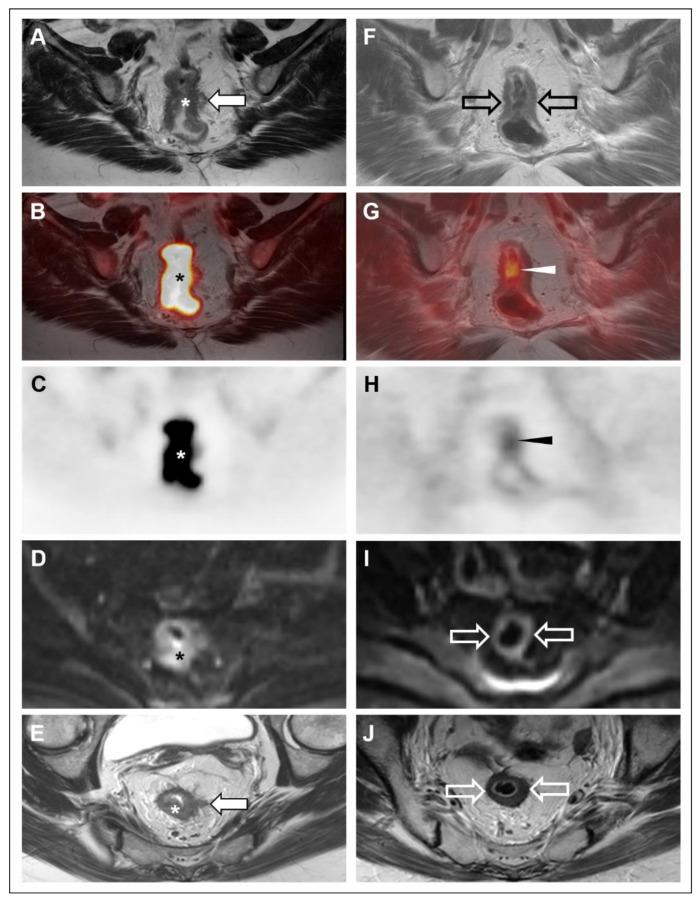
53-year-old woman with rectal adenocarcinoma (Subject #3) underwent FDG-PET/MRI at initial staging (**A**–**E**) and following TNT (**F**–**J**). Oblique coronal high-resolution coronal T2-weighted images (**A**,**F**); oblique coronal high-resolution coronal T2-weighted images with PET fusion (**B**,**G**), oblique coronal attenuation-corrected PET images (**C**,**H**), axial diffusion-weighted images (b = 1000 s/mm^2^) (**D**,**I**), oblique axial high-resolution T2-weighted images (**E**,**J**) are shown. At initial staging, a hypermetabolic, diffusion-restricting mid-rectal mass (*) with tumor extension into the mesorectal fat (closed arrows) was noted, along with several suspicious mesorectal nodes (not shown). Following TNT, there was no definite residual disease on MRI (open arrows). Both readers assigned an mrTRG of 1–2. However, PET showed residual focal hypermetabolism (arrowheads) in the treated area. Both readers assigned a pmrTRG of 2 based on possible residual tumor on PET. Subsequent lower endoscopy revealed a stricture of the treated segment that could not be traversed, though biopsies of the accessible portions were benign. Based on the suspicion of residual disease on the PET, the patient underwent a low anterior resection, with surgical pathology revealing residual disease (ypT2 ypN0).

**Figure 3:**
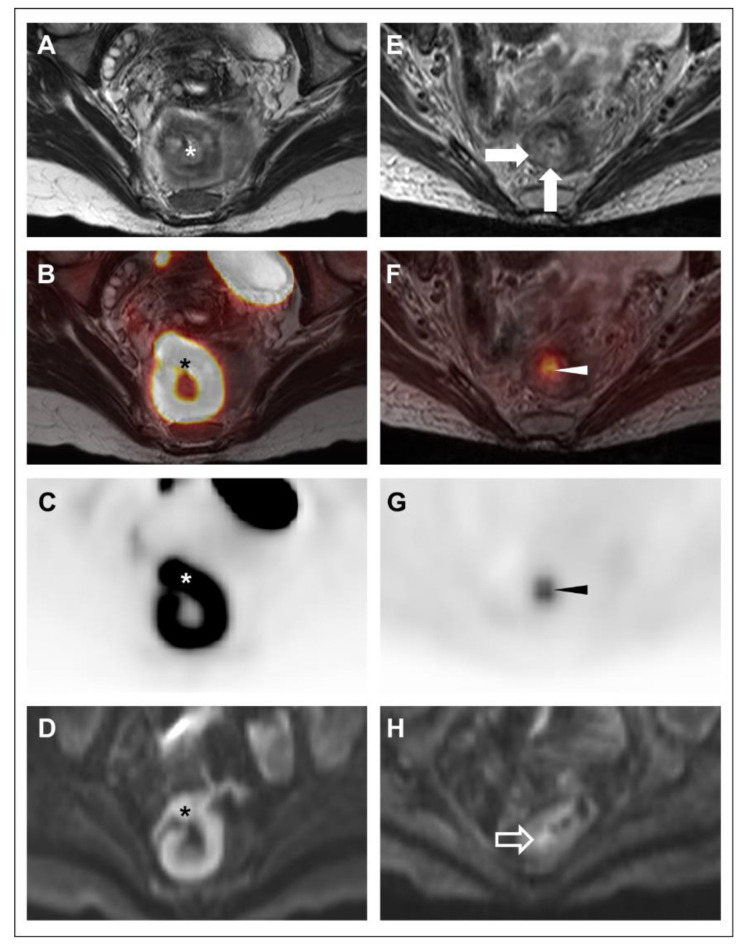
39-year-old woman with rectal adenocarcinoma (Subject #7) underwent FDG-PET/MRI at initial staging (**A**–**D**) and following TNT (**E**–**H**). Oblique axial high-resolution coronal T2-weighted images (**A**,**E**); oblique axial high-resolution coronal T2-weighted images with PET fusion (**B**,**F**), oblique axial attenuation-corrected PET images (**C**,**G**), and axial diffusion-weighted images (b = 1000 s/mm^2^) (**D**,**H**) are shown. At initial staging, a hypermetabolic, diffusion-restricting high rectal mass (*) was noted. There was no definite T3 disease, though assessment was limited by motion artifact and partial intussusception of the lesion. No suspicious lymph nodes were identified. Based on the limitations in T-stage determination, the patient underwent TNT. On restaging, both readers suspected some residual disease on MRI based on nodular T2-intermediate wall-thickening (closed arrows) and focal diffusion restriction (open arrow). Both readers assigned an mrTRG of 3. On the PET, there was a clear focus of hypermetabolism (arrowheads) in this same area, and both readers assigned a pmrTRG of 3. Although residual disease was already suspected based on the MRI alone, both readers reported a substantial increase in confidence based on the PET findings. Endoscopic biopsy confirmed residual disease, and the patient underwent resection with pathology showing ypT2 ypN0 disease.

Although FDG-PET/MRI resulted in just two changes in the consensus interpretation of disease status (i.e., mrTRG 1–2 to pmrTRG 2–3) at the first restaging time point (F/U #1), both readers reported that the FDG-PET component provided added value in 6 of 7 cases. The reader-provided explanations of added value for FDG-PET were as follows for both readers: greater confidence in tumor regression (n = 2); focus of possible/definite residual disease not seen on MRI alone (n = 2, see Figure 2); larger extent of definite residual disease than seen on MRI alone (n = 1); greater confidence in residual disease suspected on MRI alone (n = 1, see Figure 3).

The 2 subjects deemed to have cCRs on the first restaging FDG-PET/MRI (F/U #1) underwent two subsequent surveillance FDG-PET/MRIs at 6 mo (F/U #2) and 12 mo (F/U #3) (Table 3). These subjects had persistent cCRs during the 12 mo follow-up period. For all four follow-up FDG-PET/MRIs, both readers reported that the FDG-PET component added value by providing greater confidence in the lack of recurrent disease.

Table 4 shows changes in the maximum SUV (absolute and relative) of the primary rectal tumor among the 7 subjects that completed the FDG-PET/MRI after TNT. The maximum SUV declined substantially for all tumors, though the degree of change did not correlate with the presence or absence of residual disease (according to the reference standard) at the initial restaging time point (F/U #1).

Table 5 shows inter-reader reliability results for mrTRG and pmrTRG designations. There was only slight agreement in mrTRG (kappa = 0.10) between readers, when assessed according to the standard five category ordinal scale. However, most of the disagreements pertained to mrTRG 1 vs. 2 designations, both of which indicate a lack of appreciable residual tumor. When reassessed in a binary mrTRG 1–2 vs. 3–5 fashion, there was substantial agreement (kappa = 0.72). There was moderate agreement (kappa = 0.56) between readers with respect to the pmrTRG designation.

## 4. Discussion

In this pilot study, we found that FDG-PET has the potential to alter clinical assessments of disease status among subjects treated with TNT prior to anticipated NOM, relative to MRI alone, with the FDG-PET component resulting in the detection of pathologically confirmed residual disease in 2 additional subjects. Furthermore, both readers noted that the FDG-PET component provided added value for 9 of 11 (82%) restaging/surveillance studies, increasing reader confidence or revealing unsuspected residual disease, relative to MRI alone. When judged according to the reference standard, the consensus pmrTRG designations had an accuracy of 100%, increasing sensitivity for residual disease after TNT without any additional false positives.

To our knowledge, this study is the first to explore the potential role of FDG-PET/MRI in managing rectal cancer patients on NOM protocols, with two subjects each completing two follow-up FDG-PET/MRIs during a one year surveillance period. NOM surveillance has typically consisted of imaging (typically pelvic MRI and torso CT), lower endoscopy, DRE, and/or CEA, although the optimal combination and frequency of follow-up studies has not been established [12]. Furthermore, data on patient adherence to these surveillance protocols are limited. A 2017 meta-analysis exploring outcomes among rectal cancer patients on NOM found that 15.7% of patients with cCRs after neoadjuvant chemoradiation had tumor regrowth within 2 years and that 95.4% of patients with tumor regrowth subsequently underwent salvage therapies [13]. Notably, in that study, some patients were found to have unresectable disease at the time of tumor regrowth recognition, suggesting that these patients may have benefited from a more sensitive surveillance strategy [13].

To this point, our study suggests that FDG-PET/MRI may improve accuracy relative to MRI alone at post-TNT restaging by increasing sensitivity for residual disease and (by extension) for tumor regrowth in patients initially deemed to have a cCR. There were two subjects with false negative mrTRG consensus assessments but true positive pmrTRG consensus assessments; one of these two subjects also had a false negative endoscopy with biopsy. Several other studies have also assessed whether FDG-PET/MRI improves restaging accuracy after neoadjuvant chemoradiation relative to MRI alone. For example, a study of 36 patients with locally advanced rectal cancer undergoing restaging after neoadjuvant chemotherapy/radiation found that FDG-PET/MRI was more sensitive than MRI alone for residual disease at the primary tumor site (100% vs. 96%) and within regional lymph nodes (90% vs. 70%) [9]. In contrast, the gains in sensitivity for residual disease in our study were exclusively at the primary tumor site rather than within regional lymph nodes. This apparent discrepancy is likely attributable to differences in study design, in that our study was not limited to locally advanced disease and included patients (3 of 7 in the TNT cohort) without evidence of nodal metastatic disease at baseline imaging.

Our study’s observed post-TNT cCR rate at the first restaging time point, based on FDG-PET/MRI assessments and lower endoscopy (2 of 7; 29%), is lower than in other studies, which have reported cCR rates in the 49–73% range [3,4,5]. For example, our previously published institutional experience with patients treated with TNT (short-course radiation therapy followed by consolidative FOLFOX chemotherapy), 43 of 86 evaluable patients (50%) had a cCR at the first restaging time point [14]. However, when FDG-PET findings are not considered, the post-TNT cCR rate in the present study was higher (3 of 7; 43%), closer to the previously published ranges. The true post-TNT cCR rate with FDG-PET/MRI is uncertain, given the small number of subjects in our study. Nonetheless, our data suggest that the cCR rates reported by prior studies might have been lower if FDG-PET/MRI had been used instead of MRI alone for post-TNT restaging.

Our study has limitations, most notably the small sample size, which precludes robust quantitative analysis. For example, analysis of data from a larger patient population might reveal that changes in SUV during TNT predict cCR, though there was no obvious trend among the 7 patients in our study that underwent restaging FDG-PET/MRI. Furthermore, FDG-PET/MRI radiomic features, which have shown promise for predicting rectal cancer treatment response, could not be assessed due to the small number of subjects [15]. There was substantial attrition from the 14 subjects undergoing initial staging FDG-PET/MRI to the 2 subjects formally starting the NOM surveillance protocol, reducing our ability to draw meaningful conclusions about the added value of FDG-PET for detecting recurrences following an initial cCR. Future studies investigating FDG-PET/MRI for this indication might benefit from enrolling subjects at the first restaging time point or even after NOM has already begun to enhance recruitment efficiency. These studies should evaluate NOM patients for up to 24–36 months following TNT to ascertain its optimal benefit in detecting relapses. Furthermore, the results of this single-institution study may not be applicable to centers using different TNT regimens or staging/restaging imaging protocols.

## 5. Conclusions

In conclusion, FDG-PET can alter clinical response assessments for patients with rectal cancer treated with TNT due to the detection of residual disease missed by MRI alone, with FDG-PET/MRI demonstrating an accuracy of 100% for cCR status in a small patient cohort. Furthermore, the FDG-PET component provided added value in over 80% of restaging cases, based on reader assessments. Future prospective studies are needed to determine whether FDG-PET improves accuracy for detecting tumor regrowth after initial cCR designations in patients on NOM protocols.

## Figures and Tables

**Table 1:** Tumor characteristics on initial staging pelvic PET/MRI.

Tumor Characteristics	All (n = 14)	TNT (n = 7)
Clinical T-stage		
cT1	0	0
cT2	5	3
cT3a	0	0
cT3b	3	1
cT3c	2	1
cT3d	1	1
cT4a	0	0
cT4b	3	1
Extramural vascular invasion		
Positive	2	1
Negative	12	6
Location in rectum (from anal verge)		
Low (0–4.9 cm)	7	4
Mid (5–9.9 cm)	5	2
High (10–15 cm)	2	1
Clinical N-stage		
cN0	6	3
cN1a	1	0
cN1b	1	1
cN1c	0	0
cN2a	2	1
cN2b	4	2

Abbreviations: TNT = total neoadjuvant therapy.

**Table 2:** First restaging PET/MRI (F/U #1) and endoscopic disease assessments with reference standards for TNT subjects (n = 7).

Subject #	1	2	3	4	5	6	7
Initial Stage	cT3b cN1b	cT3c cN2b	cT3d cN2a	cT2 cN0	cT4b cN1b	cT2 cN0	cT2 cN0
mrTRG—F/U #1							
Reader 1	1	2	1	3	3	2	3
Reader 2	3	1	2	3	3	1	3
Consensus	1	1	2	3	3	2	3
Tumor status (MRI)—F/U #1							
Reader 1	−	−	−	+	+	−	+
Reader 2	+	−	−	+	+	−	+
Consensus	−	−	−	+	+	−	+
Node status (MRI)—F/U #1							
Reader 1	−	−	−	−	−	−	−
Reader 2	−	−	−	−	−	−	−
Consensus	−	−	−	−	−	−	−
pmrTRG—F/U #1							
Reader 1	1	1	2	3	3	1	3
Reader 2	3	2	2	3	3	1	3
Consensus	1	2	2	3	3	1	3
Tumor status (PET/MRI)—F/U #1							
Reader 1	−	−	+	+	+	−	+
Reader 2	+	+	+	+	+	−	+
Consensus	−	+	+	+	+	−	+
Node status (PET/MRI)—F/U #1							
Reader 1	−	−	−	−	−	−	−
Reader 2	−	−	−	−	−	−	−
Consensus	−	−	−	−	−	−	−
Added value of PET—F/U #1							
Reader 1	Y	Y	Y	N	Y	Y	Y
Reader 2	Y	Y	Y	N	Y	Y	Y
Consensus	Y	Y	Y	N	Y	Y	Y
Endoscopy +/− biopsy—F/U #1	−	+	−	−	+	−	+
Reference standard	F/U *	Path.	Path.	Path.	Path.	F/U *	Path.
Reference stage (post-TNT)	cT0 cN0	ypT2 ypN0	ypT2 ypN0	ypT1 ypN0	ypT2 ypN0	cT0 cN0	ypT2 ypN0
Index test adjudication—F/U #1							
Endoscopy +/− biopsy	TN	TP	FN	FN	TP	TN	TP
mrTRG consensus	TN	FN	FN	TP	TP	TN	TP
pmrTRG consensus	TN	TP	TP	TP	TP	TN	TP

* No evidence of disease on imaging (including two FDG-PET/MRIs) and endoscopy for 12 months after initial restaging PET/MRI. Abbreviations: +/− = positive/negative, FN = false negative, F/U = follow-up, mrTRG = MRI tumor regression grade, TNT = total neoadjuvant therapy, Path. = pathology from surgical resection, pmrTRG = PET/MRI tumor regression grade, TN = true negative, Y/N = yes/no.

**Table 3:** Subsequent restaging PET/MRI disease assessments for TNT subjects on nonoperative management (n = 2).

Subject #	1	6
Initial Stage	cT3b cN1b	cT2 cN0
mrTRG—F/U #2		
Reader 1	2	2
Reader 2	2	1
Consensus	2	1
pmrTRG—F/U #2		
Reader 1	1	1
Reader 2	1	1
Consensus	1	1
Added value of PET—F/U #2		
Reader 1	Y	Y
Reader 2	Y	Y
Consensus	Y	Y
Endoscopy +/− biopsy—F/U #2	−	−
mrTRG—F/U #3		
Reader 1	2	2
Reader 2	2	1
Consensus	2	1
pmrTRG—F/U #3		
Reader 1	1	1
Reader 2	1	1
Consensus	1	1
Added value of PET—F/U #3		
Reader 1	Y	Y
Reader 2	Y	Y
Consensus	Y	Y
Endoscopy +/− biopsy—F/U #3	−	−

Abbreviations: +/− = positive/negative, F/U = follow-up, mrTRG = MRI tumor regression grade, Y/N = yes/no, pmrTRG = PET/MRI tumor regression grade.

**Table 4:** SUVs for primary tumor at initial staging and first restaging time points for TNT subjects (n = 7).

Subject #	1	2	3	4	5	6	7
Initial Stage	cT3b cN1b	cT3c cN2b	cT3d cN2a	cT2 cN0	cT4b cN1b	cT2 cN0	cT2 cN0
Maximum SUV—initial staging	21.2	15.7	14.3	21.9	20.3	4.2	18.9
Maximum SUV—F/U #1	2.9	4.7	3.6	2.0	7.2	1.9	2.0
∆ SUV (absolute)	−18.3	−11.0	−10.7	−19.9	−13.1	−2.3	−16.9
∆ SUV (relative)	−86%	−70%	−75%	−91%	−65%	−55%	−89%
Reference standard	F/U *	Path.	Path.	Path.	Path.	F/U *	Path.
Reference stage (post-TNT)	cT0 cN0	ypT2 ypN0	ypT2 ypN0	ypT1 ypN0	ypT2 ypN0	cT0 cN0	ypT2 ypN0

* No evidence of disease on imaging (including two FDG-PET/MRIs) and endoscopy for 12 months after initial restaging PET/MRI. Abbreviations: F/U = follow-up, Path. = pathology from surgical resection, SUV = standardized uptake value.

**Table 5:** Inter-reader reliability analysis of mrTRG and pmrTRG scores at F/U #1.

**mrTRG**	R2: 1	R2: 2		R2: 3		R2: 4		R2: 5
R1: 1	0	1		1		0		0
R1: 2	2	0		0		0		0
R1: 3	0	0		3		0		0
R1: 4	0	0		0		0		0
R1: 5	0	0		0		0		0
kappa = 0.10								
**mrTRG**	R2: 1–2				R2: 3–5			
R1: 1-2	3				1			
R1: 3-5	0				3			
kappa = 0.72								
**pmrTRG**	R2: 1		R2: 2				R2: 3	
R1: 1	1		1				1	
R1: 2	0		1				0	
R1: 3	0		0				3	
kappa = 0.56								

Abbreviations: F/U = follow-up, mrTRG = MRI tumor regression grade, pmrTRG = PET/MRI tumor regression grade, R1 = reader 1, R2 = reader 2.

## Data Availability

The data presented in this study are available on request from the corresponding author. The data are not publicly available due to privacy concerns.
